# Obese adolescents have higher risk for severe lower extremity fractures after falling

**DOI:** 10.1007/s00383-023-05524-9

**Published:** 2023-07-19

**Authors:** Nicolas Gonzalez, Jeffry Nahmias, Sebastian Schubl, Lourdes Swentek, Brian R. Smith, Ninh T. Nguyen, Areg Grigorian

**Affiliations:** Irvine Medical Center, Department of Surgery, Division of Trauma, Burns and Surgical Critical Care, University of California, 3800 Chapman Ave, Suite 6200, Orange, CA 92868-3298 USA

**Keywords:** Adolescent obesity, Falls, Lower extremity fractures

## Abstract

**Introduction:**

Reports vary on the impact of obesity on the incidence of lower extremity fractures after a fall. We hypothesized that obese adolescents (OA) presenting after a fall have a higher risk of any and severe lower extremity fractures compared to non-OAs.

**Methods:**

A national database was queried for adolescents (12–17 years old) after a fall. Primary outcome included lower extremity fracture. Adolescents with a body mass index (BMI) ≥ 30 (OA) were compared to adolescents with a BMI < 30 (non-OA).

**Results:**

From 20,264 falls, 2523 (12.5%) included OAs. Compared to non-OAs, the rate of any lower extremity fracture was higher for OAs (51.5% vs. 30.7%, p < 0.001). This remained true for lower extremity fractures at all locations (all p < 0.05). After adjusting for sex and age, associated risk for any lower extremity fracture (OR 2.41, CI 2.22–2.63, p < 0.001) and severe lower extremity fracture (OR 1.31, CI 1.15–1.49, p < 0.001) was higher for OAs. This remained true in subset analyses of ground level falls (GLF) and falls from height (FFH) (all p < 0.05).

**Conclusions:**

Obesity significantly impacts adolescents’ risk of all types of lower extremity fractures after FFH or GLF. Hence, providers should have heightened awareness for possible lower extremity fractures in OAs.

**Level of evidence:**

IV.

## Introduction

In the United States, childhood obesity is a growing epidemic with nearly 20% of children classified as obese based on body mass index (BMI) [1]. Furthermore, obese adolescents (OAs) have greater long-term risk of developing chronic diseases such as diabetes and cardiovascular disease, as well as increased risk of musculoskeletal injuries [2–5].

The impact of obesity on clinical outcomes and injury patterns in adult trauma patients has been well studied. Obesity has been linked with an increased risk of mortality, multisystem organ failure, acute renal failure, and increased intensive care unit (ICU) days following trauma [6–9]. While a linear association between higher BMI and increased risk of falling has been identified in adult patients, there are mixed reports about the impact of obesity on the incidence of extremity fractures [10]. There is even less known about adolescent trauma patients and obesity particularly after a fall.

While most ground level falls (GLFs) occur in the elderly [10, 11], adolescent patients can suffer injuries from GLFs or falls from height (FFH). In fact, previous studies have demonstrated that OAs, in comparison to non-obese adolescents (non-OAs), sustain more severe fractures, present with higher injury severity scores (ISS), and are at increased risk of death following all traumatic injuries in general [12–16]. Additionally, OAs have been demonstrated to have a significantly higher risk of lower extremity injuries than upper extremity injuries [17, 18]. However, there are no high-quality studies evaluating the risk of lower extremity fractures in OAs after a fall. Thus, we hypothesized that OAs presenting after a fall have a higher risk of any and severe lower extremity fractures compared to non-OAs. We also performed subset analyses for adolescents presenting after a GLF and FFH.

## Methods

This study was deemed exempt, and a waiver of consent was granted for use of a de-identified national database. We utilized the Trauma Quality Improvement Program (TQIP) database, a quality improvement initiative incorporating data submitted by participating trauma centers across the United States [19]. The TQIP database was queried from 2017 to 2019 to identify adolescents (12–17-years-old) presenting after any fall (GLF or FFH). All trauma mechanisms in TQIP are defined by a corresponding event code (e-code). GLF was defined by any event code which indicated “Fall on same level” while FFH was defined by e-codes indicating “Fall from height.” We excluded all patients with missing data regarding height and weight. Two groups were compared: OAs with a BMI ≥ 30 kg/m^2^ and non-OAs with a BMI < 30 kg/m^2^. Patient demographic information was collected along with vitals on admission. Pre-hospital comorbidities included attention deficit/hyperactivity disorder (ADHD), diabetes mellitus, hypertension, smoking status, and substance abuse. The injury profile included ISS, fractures of the head, spine, ribs, upper extremity, pelvis, and lower extremity as well as solid organ injuries (i.e. kidney, liver, spleen).

The primary outcome was a lower extremity fracture. A severe fracture was defined by abbreviated injury scale (AIS) grade ≥ 3. Lower extremity fractures were further categorized based on location including fracture of the femur, knee, tibia, fibula, ankle, and foot. We performed subset analyses on patients presenting after a GLF and FFH. Other measured outcomes included the total hospital length of stay (LOS), ICU LOS, ventilator days, and mortality. Additionally, inpatient complications analyzed included deep vein thrombosis (DVT), unplanned intubation, acute kidney injury (AKI), and ventilator acquired pneumonia (VAP).

A Mann–Whitney U test was used to compare continuous variables and chi-square to compare categorical variables. Categorical data was reported as percentages and continuous data was reported as medians with interquartile range or as means with standard deviation. A multivariable logistic regression model was then used for further analysis controlling for variables including age and sex. These variables were chosen based on author consensus as they are characteristics that can be identified in the pre-hospital setting and are associated with differences in bone mineral density, bone size and bone accrual as influenced by hormonal effects on bone metabolism [4] and thus may affect rates of lower extremity fractures. The adjusted risk for lower extremity fractures was reported with odds ratios (OR) and 95% confidence intervals (CI). All p-values were two-sided with a statistical significance level of < 0.05. All analyses were performed with IBM SPSS Statistics for Windows (Version 28, IBM Corp., Armonk, NY).

## Results

### Demographics of adolescent patients presenting following a fall

From 20,264 adolescent falls, 2,523 (12.5%) included OAs. The median BMI was 34 kg/m^2^ in the OA group versus 21 kg/m^2^ in the non-OA group (p < 0.001). On presentation, the OA group had higher rates of diabetes (1.4% vs. 0.5%, p < 0.001), hypertension (0.9% vs. 0.2%, p < 0.001), and mental/personality disorders (6.0% vs. 3.6%, p < 0.001). However, OAs had a lower incidence of severe trauma (ISS > 15) (4.9% vs. 6.8%, p < 0.001) (Table 1).

**Table 1:** Demographics and admission characteristics of adolescent trauma patients following a fall stratified into non-obese and obese adolescents

Characteristic	Non-OA (n = 17,741)	OA (n = 2523)	p-value
Age, year, mean (standard deviation)	13.98 (1.38)	14.12 (1.36)	< 0.001
Male, n (%)	13,308 (75.0%)	1796 (71.2%)	< 0.001
Body mass index, median (IQR)	21 (19, 24)	34 (32, 38)	< 0.001
Comorbidities, n (%)			
ADHD	1190 (6.7%)	189 (7.5%)	0.14
Bleeding disorder	47 (0.3%)	8 (0.3%)	0.63
Congenital anomalies	316 (1.8%)	57 (2.3%)	0.09
COPD	106 (0.6%)	27 (1.1%)	0.006
Current smoker	148 (0.8%)	31 (1.2%)	0.046
Diabetes mellitus	85 (0.5%)	34 (1.4%)	< 0.001
Functionally dependent health status	90 (0.5%)	30 (1.2%)	< 0.001
Hypertension	40 (0.2%)	23 (0.9%)	< 0.001
Mental/Personality disorders	635 (3.6%)	151 (6.0%)	< 0.001
Substance abuse disorder	114 (0.6%)	17 (0.7%)	0.85
Vitals on admission, n (%)			
GCS ≤ 8	324 (2.0%)	33 (1.4%)	0.07
Hypotensive (SBP < 90 mmHg)	116 (0.7%)	9 (0.4%)	0.08
ISS > 15	1208 (6.8%)	124 (4.9%)	< 0.001
Tachycardia (HR > 120/min)	888 (5.1%)	117 (7.2%)	< 0.001
Tachypnea (RR > 22/min)	2804 (16.3%)	408 (16.7%)	0.61

*OA* Obese adolescent (body mass index ≥ 30 kg/m^2^), *non-OA* non obese adolescent (body mass index < 30 kg/m^2^), *IQR* Interquartile range, *ADHD* Attention deficit disorder/attention deficit hyperactivity disorder, *COPD* Chronic obstructive pulmonary disease, *GCS* Glasgow coma scale, *SBP* Systolic blood pressure, *ISS* Injury severity score, *HR* Heart rate, *RR* Respiratory rate

### Types of injuries sustained as a result of a fall in non-OAs vs. OAs

OAs had a higher rate of lower extremity fractures (51.5% vs. 30.7%, p < 0.001) and severe lower extremity fractures (12.7% vs. 10.1%, p < 0.001) compared to non-OAs. This injury association remained true in all lower extremity fracture locations: femur (9.6% vs. 8.4%, p = 0.047), knee (1.0% vs. 0.5%, p = 0.002), tibia (31.3% vs. 18.4%, p < 0.001), fibula (16.1% vs. 9.2%, p < 0.001), ankle (9.0% vs. 2.8%, p < 0.001), and foot (2.6% vs. 1.8%, p = 0.01). In a subset analysis of GLFs, OAs continued to have a higher rate of lower extremity fractures (55.4% vs. 33.8%, p < 0.001) and severe lower extremity fractures (13.7% vs. 9.9%, p < 0.001) compared to non-OAs, with similarly increased rates at all lower extremity fracture locations: femur (12.1% vs. 8.9%, p < 0.001), knee (1.2% vs. 0.6%, p = 0.02), tibia (33.9% vs. 21.6%, p < 0.001), fibula (14.2% vs. 9.3%, p < 0.001), ankle (7.4% vs. 2.5%, p < 0.001), and foot (1.4% vs. 0.7%, p = 0.02). Furthermore, in a subset analysis of FFH, OAs had a higher rate of lower extremity fractures (46% vs. 26.4%, p < 0.001) and severe lower extremity fractures (13.2% vs. 8.6%, p < 0.001), with similarly increased rates at all lower extremity fracture locations: femur (7.6% vs. 6.3%, p = 0.18), knee (1.1% vs. 0.5%, p = 0.03), tibia (27.2% vs. 15%, p < 0.001), fibula (16.5% vs. 9%, p < 0.001), ankle (9.9% vs. 3.1%, p < 0.001), and foot (4.4% vs. 3.3%, p = 0.1) (Table 2). Compared to non-OAs, OAs suffered lower rates of solid organ injuries: spleen (1.0% vs. 2.8%, p < 0.001), lung (1.7% vs. 3.4%, p < 0.001), liver (0.8% vs. 1.3%, p = 0.02), and kidney (0.8% vs. 1.9%, p < 0.001) (Table 3).

**Table 2:** Overall outcomes and subset analyses of falls from height and ground level falls of lower extremity fracture patterns of adolescents following a fall stratified by obesity status

Characteristic, n (%)	Non-OA (n = 17,741)	OA (n = 2523)	p-value
Lower extremity fracture	5449 (30.7%)	1299 (51.5%)	< 0.001
Severe lower extremity fracture	1791 (10.1%)	320 (12.7%)	< 0.001
Femur	1492 (8.4%)	242 (9.6%)	0.047
Knee	88 (0.5%)	25 (1.0%)	0.002
Tibia	3260 (18.4%)	789 (31.3%)	< 0.001
Fibula	1641 (9.2%)	406 (16.1%)	< 0.001
Ankle	500 (2.8%)	226 (9.0%)	< 0.001
Foot	324 (1.8%)	65 (2.6%)	0.01
Fall from height			
Lower extremity fracture	1386 (26.4%)	365 (46%)	< 0.001
Severe lower extremity fracture	454 (8.6%)	105 (13.2%)	< 0.001
Femur	331 (6.3%)	60 (7.6%)	0.18
Knee	26 (0.5%)	9 (1.1%)	0.03
Tibia	787 (15%)	216 (27.2%)	< 0.001
Fibula	471 (9%)	131 (16.5%)	< 0.001
Ankle	164 (3.1%)	79 (9.9%)	< 0.001
Foot	172 (3.3%)	35 (4.4%)	0.1
Ground level fall			
Lower extremity fracture	2464 (33.8%)	593 (55.4%)	< 0.001
Severe lower extremity fracture	722 (9.9%)	147 (13.7%)	< 0.001
Femur	646 (8.9%)	130 (12.1%)	< 0.001
Knee	42 (0.6%)	13 (1.2%)	0.02
Tibia	1570 (21.6%)	363 (33.9%)	< 0.001
Fibula	675 (9.3%)	152 (14.2%)	< 0.001
Ankle	185 (2.5%)	79 (7.4%)	< 0.001
Foot	52 (0.7%)	15 (1.4%)	0.02

*OA* Obese adolescent (body mass index > 30 kg/m^2^), *non-OA* non obese adolescent (body mass index < 30 kg/m^2^)

**Table 3:** Injury type in adolescent trauma patients presenting following a fall stratified by obesity classification

Injury type, n (%)	Non-OA (n = 17,741)	OA (n = 2523)	p-value
Kidney	330 (1.9%)	21 (0.8%)	< 0.001
Liver	236 (1.3%)	20 (0.8%)	0.02
Lung	610 (3.4%)	44 (1.7%)	< 0.001
Rib	274 (1.5%)	27 (1.1%)	0.07
Spleen	496 (2.8%)	26 (1.0%)	< 0.001

*OA* Obese adolescent (body mass index ≥ 30 kg/m^2^), *non-OA* non obese adolescent (body mass index < 30 kg/m^2^)

### Other measured outcomes of non-OAs vs. OAs following a fall

OAs had increased mean hospital LOS (3.19 days vs. 2.93 days, p < 0.001) and ICU LOS (4.10 days vs. 3.22 days, p = 0.03), as well a higher rate of VAP (0.1% vs. 0%, p = 0.02). The risk of all other measured in-hospital complications including mortality (p = 0.45) was similar between the two groups (Table 4).

**Table 4:** Clinical outcomes and complications in adolescent trauma patients presenting following a fall stratified by obesity status

Outcome	Non-OA (n = 17,741)	OA (n = 2523)	p-value
LOS, days, mean (standard deviation)	2.93 (4.11)	3.19 (3.61)	< 0.001
ICU LOS, days, mean (standard deviation)	3.22 (4.21)	4.10 (4.99)	0.03
Ventilator, days, mean (standard deviation)	3.8 (5.8)	5.6 (7.7)	0.18
Death after trauma, n (%)	33 (0.2%)	3 (0.1%)	0.45
Complications, n (%)	99 (0.6%)	17 (0.7%)	0.47
Acute kidney injury	1 (0.0%)	1 (0.0%)	0.11
Acute respiratory distress syndrome	4 (0.0%)	0 (0.0%)	0.45
Cardiac arrest with CPR	12 (0.1%)	2 (0.1%)	0.84
CAUTI	4 (0.0%)	1 (0.0%)	0.61
CLABSI	1 (0.0%)	0 (0.0%)	0.71
Deep SSI	3 (0.0%)	0 (0.0%)	0.51
Deep vein thrombosis	5 (0.0%)	2 (0.1%)	0.2
Extremity compartment syndrome	28 (0.2%)	6 (0.2%)	0.36
Myocardial infarction	0	0	–
Organ/space SSI	3 (0.0%)	0 (0.0%)	0.51
Osteomyelitis	0	0	–
Pressure ulcer	15 (0.1%)	3 (0.1%)	0.59
Pulmonary embolism	2 (0.0%)	0 (0.0%)	0.59
Severe sepsis	2 (0.0%)	0 (0.0%)	0.59
Stroke/CVA	1 (0.0%)	0 (0.0%)	0.71
Superficial incision SSI	3 (0.0%)	1 (0.0%)	0.45
Unplanned admission to ICU	37 (0.2%)	5 (0.2%)	0.91
Unplanned intubation	13 (0.1%)	4 (0.2%)	0.17
Unplanned return to OR	26 (0.2%)	2 (0.1%)	0.39
Ventilator-associated pneumonia	4 (0.0%)	3 (0.1%)	0.02

*OA* Obese adolescent (body mass index ≥ 30 kg/m^2^), *non-OA* non obese adolescent (body mass index < 30 kg/m^2^), *LOS* Length of stay, *ICU* Intensive Care Unit, *CPR* Cardiopulmonary resuscitation, *CAUTI* Catheter-associated urinary tract infection, *CLABSI* Central line-associated bloodstream infection, *SSI* Surgical site infection, *OR* Operating room, *CVA* Cerebral vascular accident

### Lower extremity fracture risk factors in adolescent patients

After adjusting for age and sex, OAs had an increased associated risk of any lower extremity fracture (OR 2.41, CI 2.22–2.63, p < 0.001) and severe lower extremity fracture (OR 1.31, CI 1.15–1.49, p < 0.001), compared to non-OAs. These findings remained true in subset analyses of GLFs and FFH: FFH lower extremity fracture (OR 2.39, CI 2.05–2.79, p < 0.001), FFH severe lower extremity fracture (OR 1.66, CI 1.32–2.09, p < 0.001), GLF lower extremity fracture (OR 2.44, CI 2.14–2.77, p < 0.001), GLF severe lower extremity fracture (OR 1.46, CI 1.21–1.76, p < 0.001) (Table 5).

**Table 5:** Multivariable* logistic regression analysis for risk of lower extremity fractures in obese adolescent patients presenting following a fall

Risk of fracture following a fall for OAs versus non-OAs	OR	CI	p-value
Lower extremity fracture all patients	2.41	2.22–2.63	< 0.001
Severe lower extremity fracture (AIS ≥ 3) all patients	1.31	1.15–1.49	< 0.001
Lower extremity fracture following fall from height	2.39	2.05–2.79	< 0.001
Severe lower extremity fracture (AIS ≥ 3) following fall from height	1.66	1.32–2.09	< 0.001
Lower extremity fracture following ground level fall	2.44	2.14–2.77	< 0.001
Severe lower extremity fracture (AIS ≥ 3) following ground level fall	1.46	1.21–1.76	< 0.001

*OA* Obese adolescent (body mass index ≥ 30 kg/m^2^), *non-OA* non obese adolescent (body mass index < 30 kg/m^2^), *AIS* Abbreviated injury scale

*Controlled for age and sex

## Discussion

Adolescent obesity is a growing epidemic with a continued expected high occurrence of falls in this age group. While there is existing literature on the effects of obesity on risk of fracture in both adult and pediatric trauma patients, to our knowledge, this is the first study evaluating the effects of obesity on the risk of lower extremity fractures in adolescent fall patients. This national analysis demonstrated that despite having a lower rate of severe trauma, OAs presenting after a GLF or FFH had an increased associated risk of lower extremity fractures and severe lower extremity fractures, compared to non-OAs. Additionally, OAs had a lower rate of solid organ injuries after a fall suggesting that obesity may serve as a protective factor for these types of injuries.

Obesity significantly impacts the risk of lower extremity fractures in adolescents following all falls. McGregor et al. demonstrated that obese children are two times more likely to have low energy mechanisms of tibia fracture, such as from a GLF, compared to non-obese children [1]. Similarly, our study’s subgroup analysis found OAs have a twofold increased risk of lower extremity fracture for GLFs and FFH, implicating obesity as a potential risk factor for lower extremity fracture regardless of mechanism of fall. Furthermore, we found that the fracture risk is ubiquitous across the lower extremity, occurring at the femur, knee, tibia, fibula, ankle, or foot. This finding is consistent with previous literature analyzing all trauma mechanisms and demonstrating a stepwise increased risk of fractures in the foot, ankle, leg, and knee with increasing BMI most pronounced in children 6–11 years old [20]. This increased risk for OAs extends beyond lower extremity fractures to other extremity injuries such as sprains/strains and dislocations [20]. We did note that the most significant differences between OAs and non-OAs occurred below the knee with OAs having a significantly higher rate of fractures to the tibia, fibula, and ankle. Several potential mechanisms could explain this observation. Firstly, the increased weight borne by the lower extremities in OAs can lead to greater mechanical stresses, making the bones more susceptible to injury even from minor trauma. This is particularly relevant for weight-bearing bones like the tibia and fibula, which are situated below the knee. Secondly, OAs often have altered biomechanics due to their increased body mass, including changes in gait and balance. These changes can increase the risk of falls and subsequent fractures. In particular, falls that are “off-axis” or at an unusual angle may be more common in this population, leading to more injuries below the knee.

Increased risk of injury may be attributed to numerous biomechanical factors related to weight distribution and alignment in the lower extremity affecting balance and ultimately contributing to negative outcomes following a fall [21]. Obese patients demonstrate altered kinetic characteristics of locomotion that can lead to poor intrinsic coordination and falling in a suboptimal position [22–24]. Additionally, previous studies have demonstrated that OAs fall with greater force from equal heights when compared to non-OAs [12]. Weight is directly proportional to energy transfer and momentum accounting for increased severity and likelihood of fracture for all falls regardless of height in OAs [12, 25]. Ultimately obesity proves deleterious to the outcomes of adolescents as obesity is associated with a greater risk of all types of lower extremity fractures regardless of whether the fall was from ground level or FFH.

Metabolic differences in OAs may contribute to a higher risk of lower extremity fracture. Studies have shown obesity to have a negative effect on attaining peak bone mass in adolescents due to the alteration of bone metabolism during this period of rapid skeletal growth [26, 27]. Obesity has been demonstrated in animal and cellular models to promote low grade inflammation. Inflammation in turn contributes to rearrangement of bone microarchitecture through bone reabsorption by reduced osteoblast and enhanced osteoclast activity. This results in increased cortical porosity and decreased trabecular thickness potentially increasing the bone’s susceptibility to fracture [14, 28, 29]. The nonspecific effect of obesity on bone, therefore contributes to greater incidence of fracture in OAs at all lower extremity locations. Ultimately, mechanical disadvantages and compromised bone microarchitecture combined may contribute to increased lower extremity fracture risk of OAs during GLFs and FFH.

In our study, we observed a small but noteworthy difference in age between obese and non-obese adolescents. This raises interesting questions about the interplay between age and obesity during adolescence, a critical period of physical growth and development. One potential explanation for this difference could be related to the onset of puberty, which varies widely and can significantly affect growth patterns and body composition. It is also important to consider lifestyle factors that change as children age. Older adolescents might be more independent in their food choices and potentially more sedentary due to increased academic pressure and decreased physical education opportunities, which might contribute to weight gain.

While obesity may be deleterious, there have been multiple studies that suggest obesity as providing an armor or shielding effect from injury [25, 30–32]. Obesity has been shown to decrease peak force of impact and increase tissue energy absorption due to increased soft tissue thickness [30]. As such, this study found that in contrast to extremity injuries, OAs had a lower rate of solid organ injuries after a fall, compared to non-OAs. With adipose tissue often localized to the abdomen in OAs, the cushion effect may not prove helpful in providing protection against extremity fractures.

As a national retrospective database study, there are inherent limitations including reporting bias, coding errors, and missing data. In addition, causation cannot be established. Also, information regarding adolescents’ level of physical activity, nutritional status, and bone density are not available in TQIP. There also exist limitations inherent to BMI as a tool for determining obesity status as BMI only takes into account weight and height, barring distinction between body fat and muscle content. Likewise, information regarding the body composition of adolescents is lacking as BMI does not provide any relevant indication of an individual’s fat distribution which may have effects on forces generated during a fall. Additionally, the circumstances preceding and ultimately causing the fall remain unknown precluding analysis of the increased risk of weight based victimization that OAs face and its effects on fracture risk [33]. Finally, TQIP is confined to index hospitalization data, thus we cannot make any statements regarding the long term outcomes of fractures in OAs.

## Conclusion

In conclusion, the influence of obesity on fracture patterns is important to understand given the increasing prevalence of obesity in adolescents. Furthermore, obese adolescents had an increased risk of all types of lower extremity fractures after both ground level falls and falls from height compared to non-obese adolescents. On the other hand, obese adolescents had a lower rate of solid organ injuries after a fall. Based on these findings, trauma providers should have increased vigilance in evaluating for lower extremity fractures in obese adolescents.
